# Supplementary material for: Integrative analysis of cell state changes in lung fibrosis with peripheral protein biomarkers
Source: EMBO Mol Med. 2021 Mar 2;13(4):e12871. doi: 10.15252/emmm.202012871 (PMC8033531; doi:10.15252/emmm.202012871)
Supplement: Supplementary file 1 — Appendix [file EMMM-13-e12871-s011.pdf]

Appendix

Integrative analysis of cell state changes in lung fibrosis with peripheral protein biomarkers

Christoph H. Mayr[1]*, Lukas M. Simon[2]*, Gabriela Leuschner[1,3], Meshal Ansari[1,2], Janine Schniering[1,4], Philipp E. Geyer[5], Ilias Angelidis[1], Maximilian Strunz[1], Pawandeep Singh[1], Nikolaus Kneidinger[3], Frank Reichenberger[6], Edith Silbernagel[6], Stephan Böhm[7], Heiko Adler[8], Michael Lindner[6,12], Britta Maurer[4], Anne Hilgendorff[9], Antje Prasse[10], Jürgen Behr[3,6], Matthias Mann[5], Oliver Eickelberg[11], Fabian J. Theis[2,#], and Herbert B. Schiller[1,#]

This PDF file includes:

Appendix Figures S1 to S7

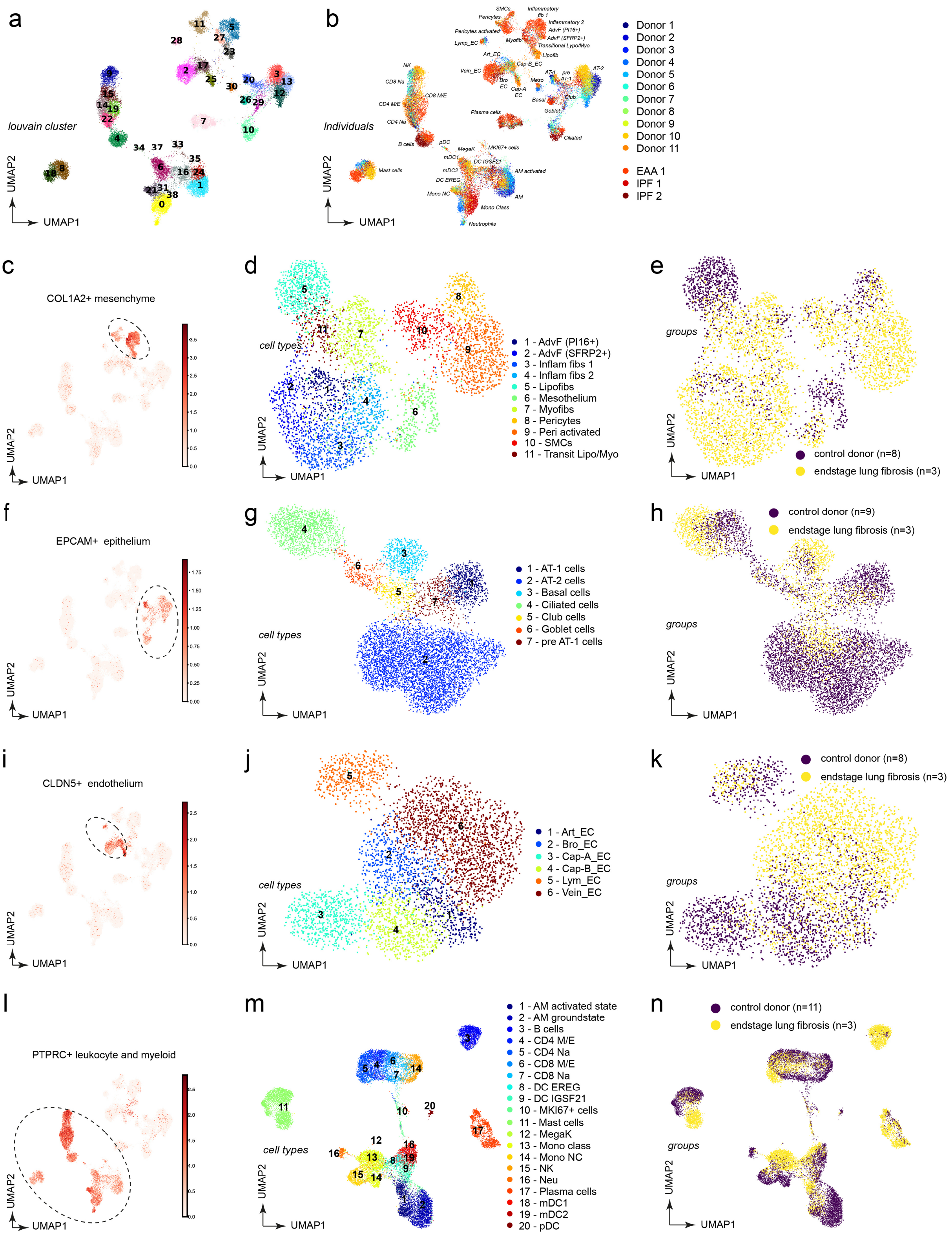

Appendix Figure S1. Clustering analysis and cell type annotation reveals 45 distinct cell type identities in human lung parenchyma. (a) UMAP embedding colored by Louvain clusters demonstrates separation of cells into major lineages. (b,c) UMAP embedding displays identified cell types, colored by individual control patients (b) and ILD patients (c). (d, g, j, m) The whole lung parenchymal dataset was split into subsets for (d) COL1A2+ mesenchymal cells, EPCAM+ epithelial cells (g), CLDN5+ endothelial cells (j) and CD45+ (gene name PTPRC) leukocytes (m). (e, h, k, n) New UMAP embeddings of the subsets demonstrate separation of cluster identities that allows for identification of cell states. (f, i, l, o) Cells colored in disease groups show origin of identified cell states.

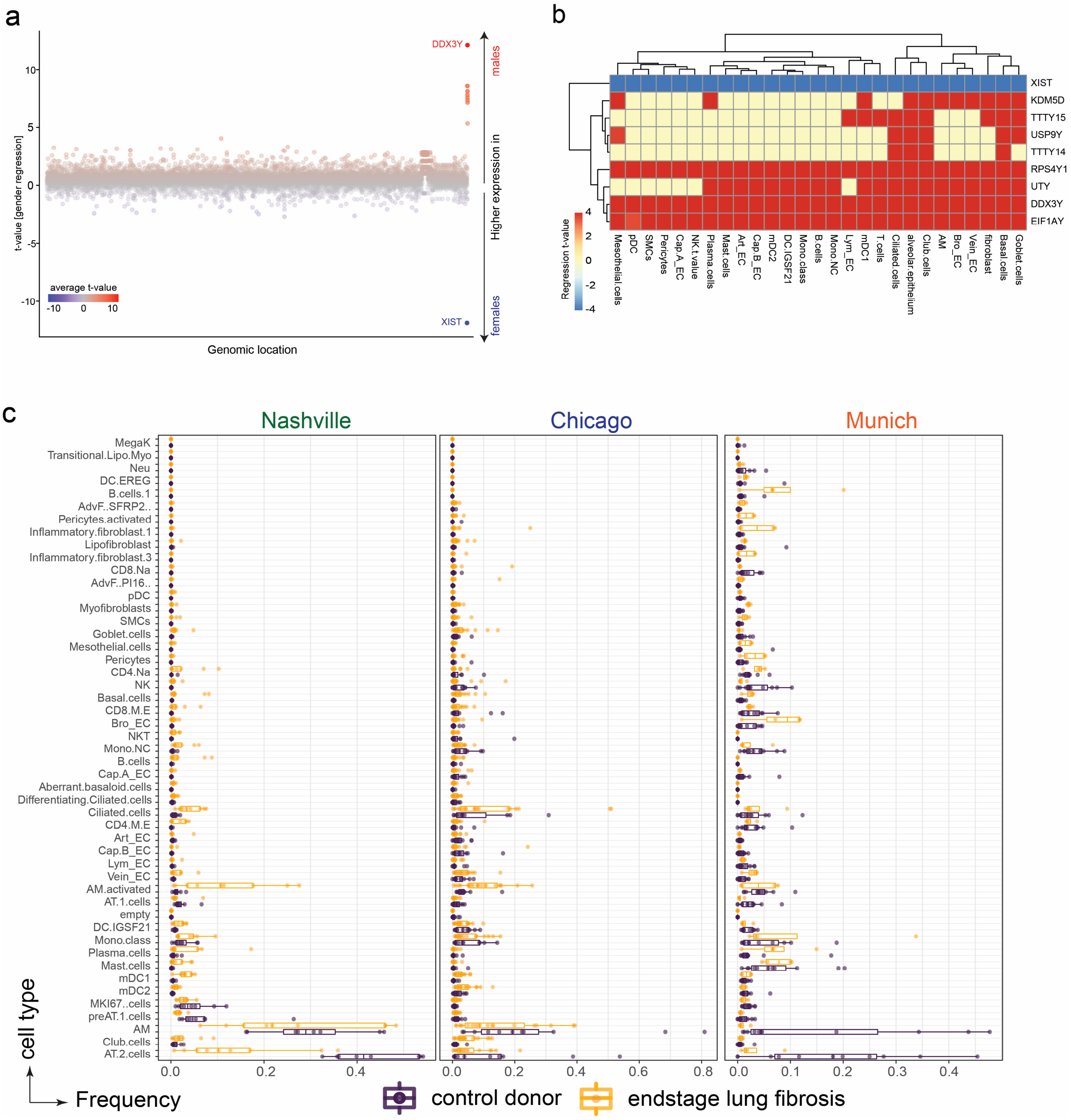

Appendix Figure S2. Differential detection and cell type frequency analyses. (a) Manhattan plot depicts the genomic location (x-axis) and average t-value across cell types (y-axis) for all genes included in the analysis. (b) Heatmap displays t-values of most significant (Multivariate regression, p-value < 1e-10) genes (rows) across cell types (columns). For both panels, red and blue colors represent high (higher expression in males) and low (higher expression in females) t-values, respectively. (c) Boxplots illustrate the frequencies (x-axis) of cell types (y-axis) across samples from ILD patients (yellow) and control donors (purple) from the three indicated cohorts.

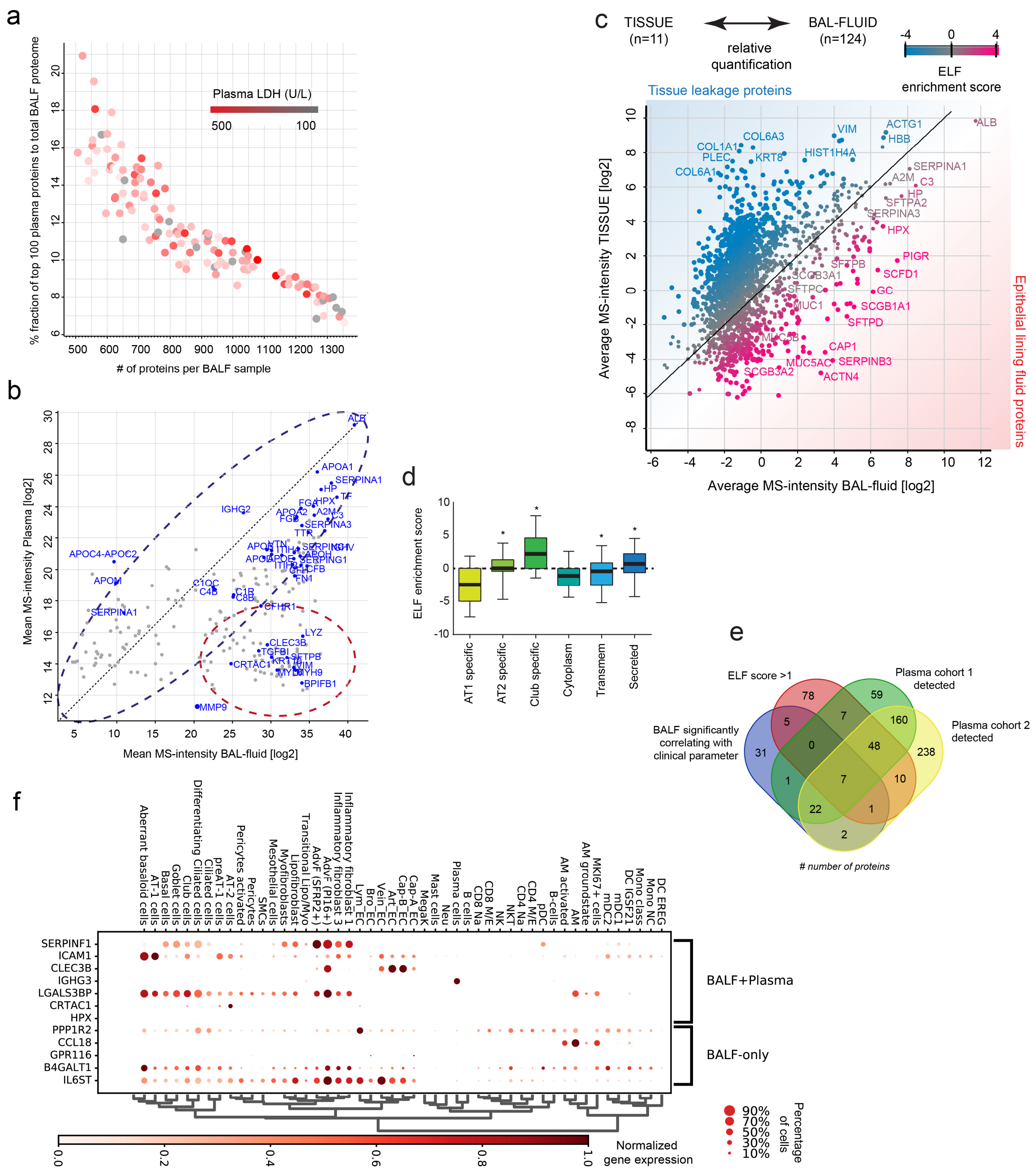

Appendix Figure S3. Defining the epithelial lining fluid proteome in a large interstitial lung disease patient cohort. (a) The fraction of plasma proteins (defined by top 100 abundance rank in plasma) is shown as a percentage of total mass fraction of all identified proteins in BALF. Note that this fraction negatively correlates with the total number of identified proteins in BALF. The colors represent plasma LDH values of individual patients. (b) The scatter plot shows mean MS-intensities of proteins identified in both plasma and BALF from all ILD patients. The red circle indicates locally enriched proteins that are more abundant in the lung compared to plasma samples. (c) Comparison of BAL fluid proteome (n=128) and ILD lung tissue proteome (n=11) allows prediction of major constituents of the epithelial lining fluid as opposed to tissue leakage proteins and identifies 199 proteins with significant enrichment in the BAL-fluid proteome. The color code shows the relative enrichment of proteins in fluid versus tissue (ELF score). (d) The box plot shows distributions of ELF enrichment scores for the indicated gene categories. (e) The Venn diagram visualizes the overlap of proteins between the significantly correlating BALF proteins (Fig. 4c) with the proteins with an ELF score greater than 1 (Fig. S3c), on the one hand, and the two plasma cohorts on the other hand (Fig. 8p). (f) Dotplot illustrates cell type specific mRNA expression patterns for proteins with an ELF score greater than 1. Both proteins that are significantly correlating BALF genes and proteins overlapping between BALF and are detected in the plasma cohorts are shown.

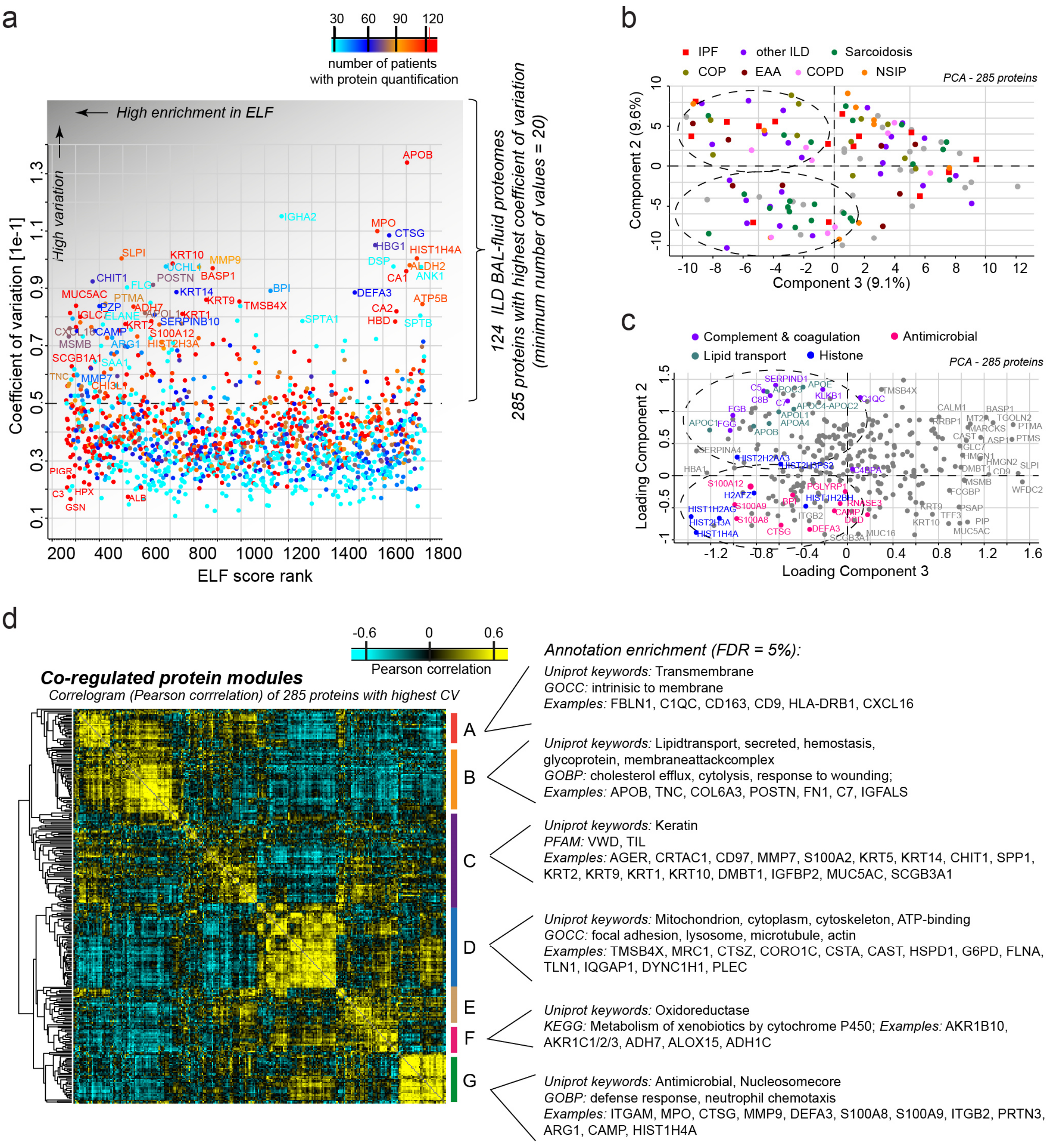

Appendix Figure S4. Co-regulated protein modules in ILD BALF proteomes. (a) Identification of 285 proteins with highest coefficient of variation and heterogeneity between patients enriched in human BALF. (b, c) Principal component analysis reveals groups of patients with similar enrichment for the indicated gene categories, irrespective of the indicated clinical diagnosis. (d) The correlogram shows the Pearson correlation of the 285 proteins with highest CV across individual patients. Enriched gene categories and examples of co-regulated proteins are shown.

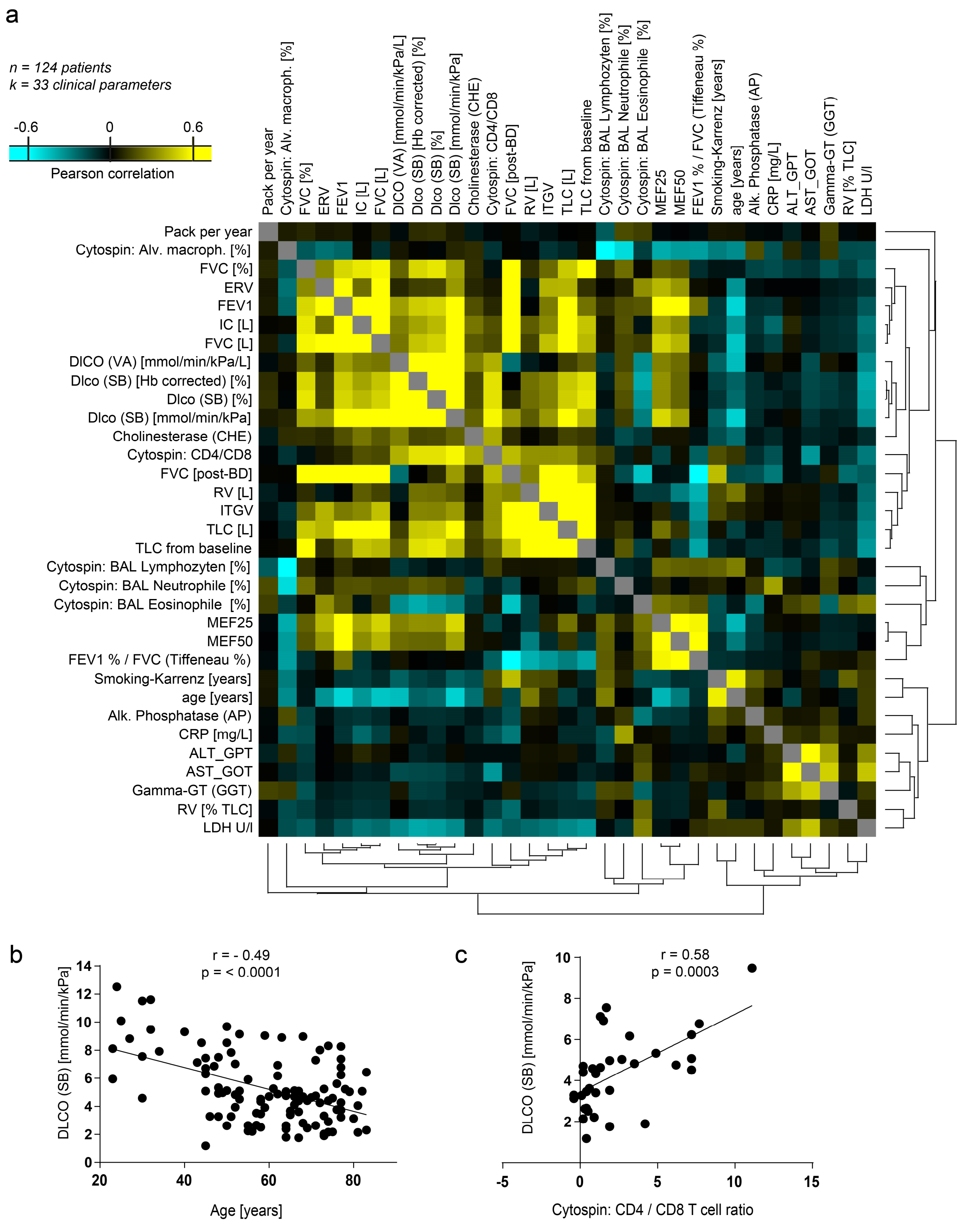

Appendix Figure S5. Correlation patterns between 33 clinical parameters in an ILD cohort. (a) Pairwise Pearson correlation values of 33 clinical parameters were grouped by hierarchical cluster analysis. (b) DLCO shows negative correlation with age in the study cohort (p<0.0001). (c) Positive correlation of CD4/CD8 ratio in BAL fluids with DLCO.

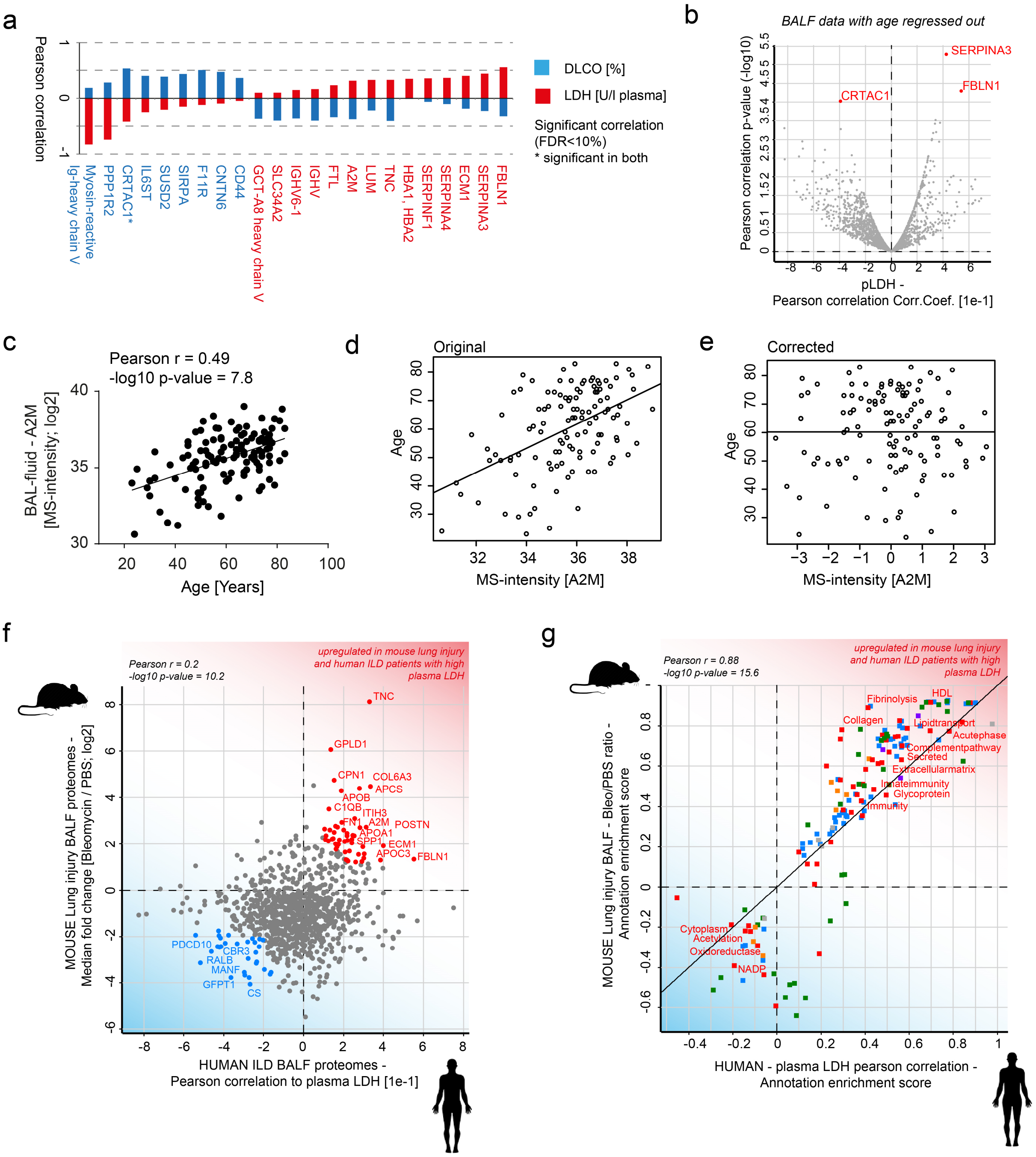

Appendix Figure S6. BALF proteins correlating with plasma LDH represent a human lung injury signature. (a) The bar graph shows the Pearson correlation values of the indicated proteins for DLCO [%] (blue) and plasma LDH (red). (c) The scatter plot shows significant correlation of alpha 2 macroglobulin (A2M) abundance (MS-intensity) in BALF with patient age. (d, e) Original (d) and age-corrected (e) correlation of A2M in BAL fluids with age. (f) The scatter plot shows the Pearson correlation of individual proteins from the human BAL fluid proteome with plasma LDH (x-axis) and the fold changes (y-axis) of the orthologous proteins in mouse lung after bleomycin injury[68]. (g) The annotation enrichment score shows a common upregulation of gene categories like acute phase, ECM, complement and innate immunity in the BAL fluids of bleomycin mice and human ILD with high plasma LDH.

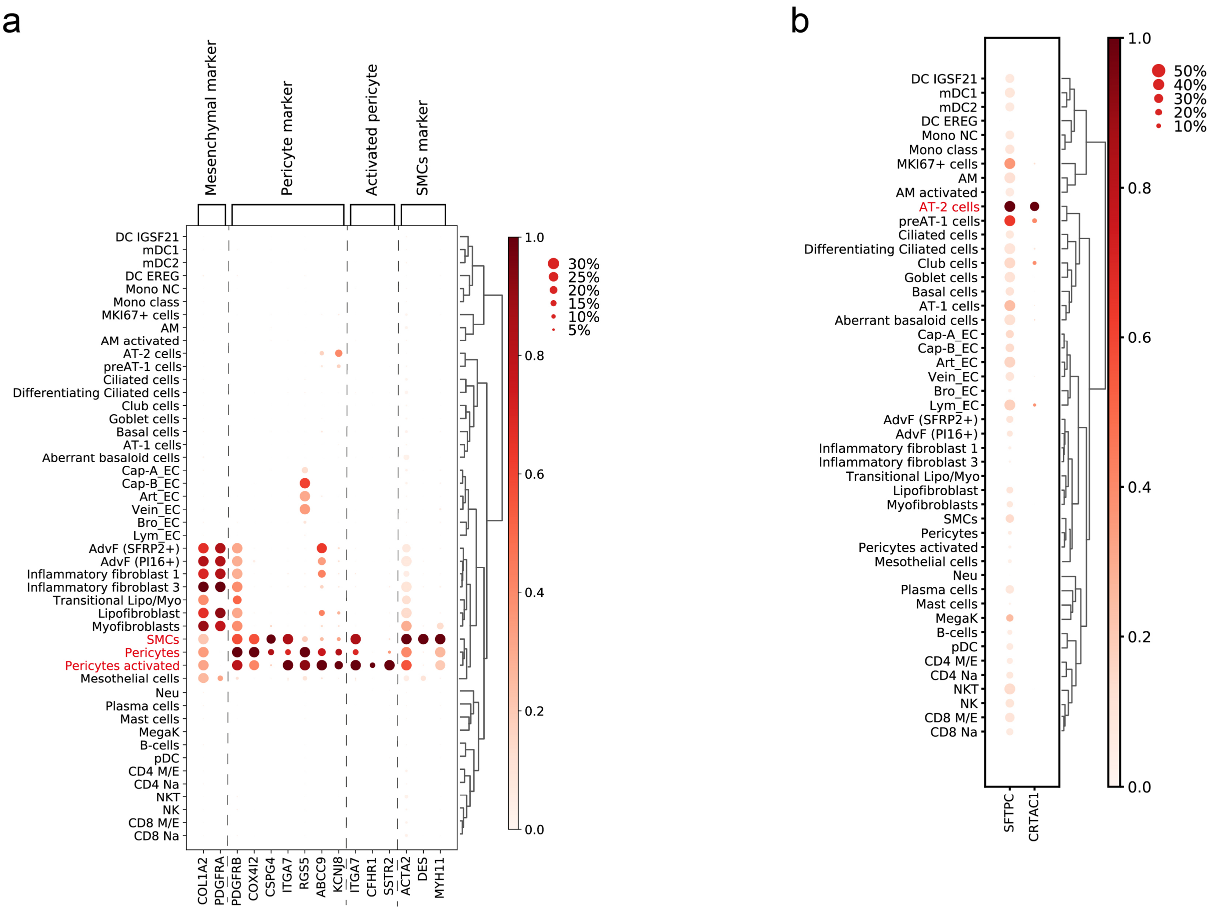

Appendix Figure S7. Cell-type specificity of CFHR1, SSTR2 and CRTAC1. (a, b) The dotplots illustrate cell type specific mRNA expression patterns for CFHR1, SSTR2 and pericyte markers (a) as well as CRTAC1 and the AT-2 cell marker SFTPC (b).
