# Supplementary material for: Integrative analysis of cell state changes in lung fibrosis with peripheral protein biomarkers
Source: EMBO Mol Med. 2021 Mar 2;13(4):e12871. doi: 10.15252/emmm.202012871 (PMC8033531; doi:10.15252/emmm.202012871)
Supplement: Supplementary file 4 — Dataset EV3 [file EMMM-13-e12871-s003.docx]

Cell types Abbreviations used in text

**COL1A2+ mesenchymal cell types**

Adventitial Fibroblasts (PI16+) AdvF (PI16+)

Adventitial Fibroblasts (SFRP2+) AdvF (SFRP2+)

Inflammatory Fibroblasts 1 Inflammatory fib 1

Inflammatory Fibroblasts 2 Inflammatory fib 1

Lipofibroblasts Lipofibs

Mesothelial cells Meso

Myofibroblasts Myofibs

Pericytes -

Pericytes Activated -

Smooth Muscle Cell SMCs

Transitional Lipofibroblasts/Myofibroblasts Transitional Lipo/Myo

**EPCAM+ epithelial cell types**

Alveolar Type-1 cells AT-1 cells

Alveolar Type-2 cells AT-2 cells

Basal cells -

Ciliated cells -

Club cells -

Goblet cells -

preAT-1 cells -

**CLDN5+ endothelial cell types**

Arterial endothelial cells Art_EC

Bronchial endothelial cells Bro_EC

Capillary endothelial cells type A Cap-A_EC

Capillary endothelial cells type B Cap-B_EC

Lymphatic endothelial cells Lym_EC

Vein endothelial cells Vein_EC

**PTPRC+ (CD45+) immune cell types**

Alveolar macrophages AM

Alveolar macrophages activated AM activated

B-cells -

CD4 memory/effector T cells CD4 M/E

CD4 naive T cells CD4 Na

CD8 memory/effector T cells CD8 M/E

CD8 naive T cells CD8 Na

Dendritic cells (EREG+) DC EREG

Dendritic cells (IGSF21+) DC IGSF21

Proliferating leukocytes MKI67+ cells

Mast cells -

Megakariocytes MegaK

Classical monocytes Mono class

Nonclassical monocyte Mono NC

Natural killer cells NK

Neutrophils Neu

Plasma cells -

Myeloid dendritic cells type 1 mDC1

Myeloid dendritic cells type 2 mDC2

Plasmacytoid dendritic cell pDC
