# Supplementary material for: Integrative analysis of cell state changes in lung fibrosis with peripheral protein biomarkers
Source: EMBO Mol Med. 2021 Mar 2;13(4):e12871. doi: 10.15252/emmm.202012871 (PMC8033531; doi:10.15252/emmm.202012871)
Supplement: Supplementary file 7 — Dataset EV5 [file EMMM-13-e12871-s012.docx]

|  | **All (n=124)** | **IPF (n=16)** | **ILD (n=25)** | **HP (n=8)** | **NSIP (n=10)** | **RB-ILD (n=3)** | **Sarcoidosis (n=22)** | **COP (n=11)** | **Non-ILD (=29)** |
| --- | --- | --- | --- | --- | --- | --- | --- | --- | --- |
| Age, years | 60.3±14.5 | 68.0±8.3 | 68.0±8.3 | 61.3±16.1 | 63.5±8.6 | 42.7±11.0 | 47.3±15.0 | 67.0±9.9 | 60.8±14.4 |
| Female gender, n (%) | 55 (44.4) | 4 (25.0) | 13 (52) | 3 (37.5) | 7 (70) | 1 (33.3) | 6 (27.3) | 7 (63.6) | 14 (48.3) |
| History of smoking | 73 (65.8)  n= 111 | 12 (85.7)  n=14 | 15 (75.0)  n=20 | 3 (50.0)  n=6 | 5 (10.0)  n=10 | 3 (100)  n=3 | 11 (52.4)  n=21 | 6 (66.7)  n=9 | 18 (64.3)  n=28 |
| Pack years | 22.3±23.1 | 40.5±24.9 | 17.5±15.8 | 20.0±28.3 | 35.7±32.2 | 25.0±8.7 | 6.8±10.2 | 27.0±20.2 | 22.2±24.6 |
| **Lung function** |  |  |  |  |  |  |  |  |  |
| FVC [l] (n=112) | 2.9±1.0 | 2.5±0.7 | 2.7±0.9 | 2.7±0.8 | 2.3±0.5 | 3.1±0.4 | 3.8±1.2 | 2.5±0.6 | 3.1±1.0 |
| FVC [% target] (n=112) | 80.0±19.8 | 71.5±17.1 | 75.8±16.6 | 77.9±21.6 | 79.5±20.6 | 71.4±4.5 | 88.1±19.3 | 80.9±22.0 | 83.9±22.4 |
| FEV1 [l] (n=112) | 2.3±0.9 | 2.1±0.7 | 2.1±0.7 | 2.2±0.7 | 1.9±0.4 | 2.4±0.8 | 3.1±1.0 | 2.0±0.6 | 2.4±1.0 |
| FEV1 %/ FVC  (Tiffeneau %) (n=112) | 81.9±11.9 | 86.7±8.6 | 81.3±8.4 | 82.9±12.4 | 88.5±14.5 | 75.0±15.8 | 84.3±7.7 | 80.1±17.3 | 77.0±13.1 |
| TLC [l] (n=110) | 5.2±1.4 | 4.5±1.1 | 5.0±1.6 | 5.2±0.6 | 4.1±0.9 | 4.9±0.4 | 5.8±1.5 | 4.8±1.0 | 5.9±1.4 |
| RV [l] (n=111) | 2.3±0.9 | 1.9±0.6 | 2.3±1.0 | 2.4±0.4 | 1.8±0.7 | 1.8±0.1 | 2.0±0.6 | 2.3±0.4 | 2.8±1.0 |
| DLCo (SB) [% target] (n=94) | 58.3±21.8 | 42.6±20.9 | 51.6±16.8 | 52.9±13.7 | 46.8±9.1 | 48.6±0.4 | 73.5±22.5 | 61.2±23.9 | 67.4±21.8 |
| **Laboratory findings** |  |  |  |  |  |  |  |  |  |
| LDH U/l (n=83) | 249.1±87.3 | 266.1±93.3 | 241.1±84 | 242.9±90.2 | 340.4±100.3 | 190.5±30.4 | 212.7±60.5 | 270.7±102.9 | 234.8±73.1 |
| CRP [mg/l] (n=85) | 16.6±27.4 | 7.3±6.1 | 21.3±37.2 | 3.0±1.4 | 8.8±8.4 | 3.4±0.6 | 10.0±20.0 | 39.8±33.6 | 20.1±30.1 |
| **BAL cytospin (n=36)** |  |  |  |  |  |  |  |  |  |
| BAL alveolar macrophages [%] | 66.9±23.0 | 71.9±24.5 | 66.1±20.6 | 38.6±19.7 | 84.3±13.1 | 92.8±2.9 | 60.4±27.5 | 49.3±27.2 | 72.8±13.5 |
| BAL lymphocytes [%] | 24.4±19.7 | 20.8±24.3 | 32.3±16.3 | 55.0±15.8 | 8.4±8.1 | 3.0±1.1 | 30.8±14.4 | 28.2±25.4 | 16.1±11.5 |
| BAL neutrophils [%] | 8.2±15.4 | 2.2±1.3 | 3.6±3.1 | 3.0±0.3 | 7.7±6.6 | 3.5 | 9.2±16.0 | 30.3±43.3 | 8.7±8.1 |
| BAL eosinophils [%] | 2.6±3.4 | 4.3±3.6 | 2.9±4.9 | 3.3±4.2 | 1.8±1.9 | 1.9±0.1 | 0.5±0.5 | 2.5±2.6 | 2.2±3.9 |
| CD4/CD8 quotient | 2.6±2.9 | 2.3±3.1 | 2.4±3.2 | 3.9±4.7 | 0.7±0.9 | 1.4±0.8 | 5.0±5.1 | 1.5±1.2 | 3.3±2.4 |

**Table S5a: Clinical characteristics of bronchoalveolar lavage fluid study cohort**

Abbreviations: idiopathic pulmonary fibrosis (IPF), interstitial lung disease (ILD), hypersensitivity pneumonitis (HP), non-specific idiopathic pneumonitis (NSIP), respiratory bronchiolitis interstitial lung disease (RB-ILD), cryptogenic organizing pneumonia (COP), bronchoalveolar lavage (BAL)

|  | **Non-ILD (=29)** | **Lung cancer (n = 11)** | **COPD (n = 9)** | **Asthma (n = 4)** | **Bronchitis (n = 2)** | **Cough (n = 3)** |
| --- | --- | --- | --- | --- | --- | --- |
| Age, years | 60.8±14.4 | 64.7±10.9 | 65.9±7.4 | 53.8±3.8 | 30.5±6.5 | 62.0±7.3 |
| Female gender, n (%) | 14 (48.3) | 6 (54.5) | 3 (27.2) | 3 (75.0) | 0 (0.0) | 2 (66.7) |
| History of smoking | 18 (64.3)  n=28 | 4 (27.3)  n=7 | 8 (77.8)  n=8 | 1 (25.0)  n=4 | 2 (100.0)  n=2 | 1 (33.3)  n=3 |
| Pack years | 22.2±24.6 | 10.8±11.0 | 39.4±24.1 | 7.5±11.3 | 6.5±3.5 | 6.7±8.9 |
| **Lung function** |  |  |  |  |  |  |
| FVC [l] | 3.1±1.0 | 2.8±0.6 | 3.0±0.6 | 3.4±0.7 | 5.4±0.5 | 2.6±0.3 |
| FVC [% target] | 83.9±22.4 | 77.3±16.6 | 82.5±15 | 89.9±17.1 | 98.6±11.6 | 94.4±9.4 |
| FEV1 [l] | 2.4±1.0 | 2.8±0.6 | 2.0±0.6 | 2.4±0.9 | 4.7±0.1 | 2.3±0.4 |
| FEV1 %/ FVC (Tiffeneau %) | 77.0±13.1 | 80.8±7.3 | 66.7±9.0 | 73.9±13.4 | 88.5±8.2 | 86.9±5.5 |
| TLC [l] | 5.9±1.4 | 5.5±1.0 | 6.4±1.0 | 6.0±1.0 | 7.9±0.2 | 4.9±0.5 |
| RV [l] | 2.8±1.0 | 2.6±0.8 | 3.4±0.8 | 2.6±0.7 | 2.6±0.3 | 2.2±0.7 |
| DLCo (SB) [% target] | 67.4±21.8 | 86.9±9.5 | 51.9±14,6 | 96.7±5.8 | 99.0±0.1 | 58.7±17.1 |
| **Laboratory findings** |  |  |  |  |  |  |
| LDH U/l | 234.8±73.1 | 201.6±26.6 | 239.7±48.5 | 275.5±68.5 | 232.5±83.5 | 281.7±104.2 |
| CRP [mg/l] | 20.1±30.1 | 6.5±4.5 | 31.7±26,8 | 29.1±39.5 | 41.2±39.2 | 91.±6.0 |
| **BAL cytospin** |  |  |  |  |  |  |
| BAL alveolar macrophages [%] | 72.8±13.5 | 69.5±6.0 | 77.2±14.1 | - | - | - |
| BAL lymphocytes [%] | 16.1±11.5 | 22.0±6.0 | 8.2±6.3 | - | - | - |
| BAL neutrophils [%] | 8.7±8.1 | 7.0±5.0 | 11.0±7.2 | - | - | - |
| BAL eosinophils [%] | 2.2±3.9 | 1.2±1.1 | 3.6±4.6 | - | - | - |
| CD4/CD8 quotient | 3.3±2.4 | 4.6±1.2 | 1.6±1.7 | - | - | - |

**Table S5b: Clinical characteristics of BALF non-ILD patients**
