# Supplementary material for: Integrative analysis of cell state changes in lung fibrosis with peripheral protein biomarkers
Source: EMBO Mol Med. 2021 Mar 2;13(4):e12871. doi: 10.15252/emmm.202012871 (PMC8033531; doi:10.15252/emmm.202012871)
Supplement: Supplementary file 12 — Dataset EV10 [file EMMM-13-e12871-s013.docx]

**Dataset EV10 – comparison of clinical data from plasma proteome cohort Munich 3 and cohort Hannover**

|  | **All**  **n=111** | **Cohort Munich 3**  **n=30** | **Cohort Hannover**  **n=81** |
| --- | --- | --- | --- |
| Age, years | 64.8±11.7 | 62.4±7.3 | 65.7±12.9 |
| FVC [% target] | 65.7±19.2 | 55.4±16.9 | 69.3±18.7 |
| DLCo (SB) [% target] | 46.6±21.4 | 27.5±17.2 | 53.8±18.3 |
| LDH [U/l] | 172.5±96.4 | 286.4±113.5 | 130.3±37.9 |
